# Domain swapping of complementarity-determining region in nanobodies produced by *Pichia pastoris*

**DOI:** 10.1186/s13568-019-0833-2

**Published:** 2019-07-15

**Authors:** Natsuko Miura, Kana Miyamoto, Yuta Ohtani, Kenshi Yaginuma, Shunsuke Aburaya, Yoshinori Kitagawa, Wataru Aoki, Mitsuyoshi Ueda

**Affiliations:** 1 Graduate School of Agriculture, Kyoto University, Kyoto, 606-8502 Japan; 2 JST, Core Research for Evolutional Science and Technology (CREST), 7 Goban-cho, Chiyoda-ku, Tokyo, 102-0076 Japan; 3 Present Address: Graduate School of Life and Environmental Sciences, Osaka Prefecture University, 1-1 Gakuen-cho, Naka-ku, Sakai, 599-8531 Japan; 4 Research Fellow of the Japan Society for the Promotion of Science, Kitashirakawa Oiwake-cho, Sakyo-ku, Kyoto, 606-8502 Japan; 5 Kyoto Integrated Science & Technology Bio-Analysis Center, 134, Chudoji Minamimachi, Simogyo-ku, Kyoto, 600-8813 Japan

**Keywords:** CDR swapping, CD4 nanobody, GFP nanobody, Relative binding ability, *Pichia pastoris*

## Abstract

Easy preparation of chimeric nanobodies with various scaffolds is important for customizing abilities of nanobodies toward practical utilization. To accomplish high-throughput production of various nanobodies, utilization of microbes is an attractive option. In the present study, various chimeric nanobodies were prepared using the methylotrophic yeast *Pichia pastoris*. We designed chimeric nanobodies with complementarity-determining regions (CDRs) against green fluorescent protein (GFP) or cluster of differentiation 4 (CD4) based on the scaffold of GFP-nanobody. FLAG-tagged chimeric nanobodies were prepared by one-step cloning and produced using *P. pastoris*. Secreted chimeric nanobodies were purified from the culture media of *P. pastoris* transformants. Relative binding abilities of purified chimeric nanobodies to GFP and CD4 was tested using a BIACORE T-200. *P. pastoris* successfully produced a high yield of FLAG-tagged chimeric nanobodies. FLAG-tagged GFP- and CD4-nanobodies were shown to specifically bind to GFP and CD4, respectively. Chimeric nanobodies, in which the CDR2 or 3 of GFP-nanobody was replaced with CDRs of CD4-nanobody, acquired the ability to bind to CD4 without binding to GFP. These results demonstrate successful production of functional chimeric nanobodies using *P. pastoris*. These results also suggest that swapping of CDRs, especially CDRs 2 or 3, potentially enables a novel method of creating nanobodies.

**Electronic supplementary material:**

The online version of this article (10.1186/s13568-019-0833-2) contains supplementary material, which is available to authorized users.

## Introduction

The heavy chain domains of *Camelidae* sp. antibodies (Hamers-Casterman et al. 1993), or nanobodies, are increasingly attracting attention for their small size and ability to bind specifically to targets. The practical applications of nanobodies range from capturing certain molecules (Beghein and Gettemans 2017) to delivering molecules to targets in vivo (Hu et al. 2017), offering attractive tools for diagnostics (De Meyer et al. 2014; Hu et al. 2017) and therapeutics (Bannas et al. 2017; Hu et al. 2017). Nanobodies are also used in in vitro molecular studies, for example for isolating proteins and protein-associated molecules (Bannas et al. 2017).

Being single-domain antibodies, nanobodies are easily produced by in vitro methods (McMahon et al. 2018) or by various host cells including bacteriophages (Arbabi Ghahroudi et al. 1997; Pardon et al. 2014), *Escherichia coli* (Conrath et al. 2001; Pardon et al. 2014), yeast cells such as *Aspergillus* sp. (Joosten et al. 2005; Okazaki et al. 2012), *Saccharomyces cerevisiae* (Gorlani et al. 2012), and *Pichia pastoris* (Ezzine et al. 2012), in addition to mammalian cells (Agrawal et al. 2012; Zhang et al. 2009). According to comparison of different methods for producing nanobodies by Frenzel and De Meyer, optimized yield of nanobodies is generally similar among Gram-negative microbes, yeast cells, and mammalian cells (Frenzel et al. 2013; De Meyer et al. 2014). Mammalian production benefits that the produced nanobodies can have mammalian post-translational modifications (Palomares et al. 2004). Microbial production of recombinant proteins is easier to test (Ferrer-Miralles and Villaverde 2013), and has been used for high-throughput screening of protein libraries (Boder and Wittrup 1997). Recently, yeast cells including the methylotrophic yeast *P. pastoris* have attracted increased attention as a host for nanobody production (Baghban et al. 2016; Pourasadi et al. 2017). In addition to being a microbe which allows high-throughput production, *P. pastoris* enables secretory production of recombinant proteins thorough folding control system of eukaryotic cells (Cereghino et al. 2002).

Various synthetic nanobodies have been reported through screening of nanobodies having random sequences. Phage display and cell surface display system enabled genotype–phenotype linking of nanobody libraries and thus enabled production and screening of large number of nanobodies (Galan et al. 2016; McMahon et al. 2018; Yan et al. 2014). Gu and colleagues reported in vitro production of DNA-barcoded nanobody library (Gu et al. 2014). In the method, random nanobody sequences are fused with random DNA barcodes and can be identified through concentration of barcodes with functional molecules followed by next-generation sequencing (NGS) analysis (Gu et al. 2014). Recently, production of peptide-barcoded nanobodies using microbial hosts has been reported, in which random nanobody sequences are fused with random peptide barcodes and can be identified by LC–MS/MS and NGS analysis (Egloff et al. 2019; Miyamoto et al. 2019). Egloff and colleagues prepared library of randomized nanobody sequences fused with peptide library produced by *E. coli* (Egloff et al. 2019). We reported secretory production of peptide-barcoded nanobodies using *P. pastoris* (Miyamoto et al. 2019). Since peptide-barcodes are smaller compared to nanobody itself, they have a little effect on binding abilities of nanobodies compared to conventional FLAG-tag composed by eight amino acids (Miyamoto et al. 2019).

Although a number of studies aim at “random” screening of nanobodies, practically it is important to select amino acids or amino acid regions to be mutated, for library preparation. Ribosome and phage display methods enable screening of up to 10^7^–10^12^ library size (Zimmermann et al. 2018), which allow limited number of amino acid residues to be changed. Nanobody has three variable regions called complementarity-determining regions (CDRs). Previously, Deschaght and colleagues showed that there are large varieties in amino acid sequence of CDRs compared to other region, via NGS analysis of 28 nanobodies (Deschaght et al. 2017). So far, CDR has been used to prepare protein libraries in various hosts including yeast cells (McMahon et al. 2018) or phage (Yan et al. 2014) in addition to in vitro methods including ribosome display (Zimmermann et al. 2018). Of three CDRs of nanobodies, it has been suggested that, in some nanobodies, CDR3 is largely responsible for specific binding to substrates (Gray et al. 2017), which has been used as a target for randomization when preparing nanobody libraries (Zimmermann et al. 2018; Moutel et al. 2016).

In practical nanobody applications, CDRs are inserted into proper scaffolds, such as universally humanized scaffolds (Vincke et al. 2009; Moutel et al. 2016) or scaffolds suitable for tissue imaging (De Groeve et al. 2010), with some integrated mutations to retain binding abilities (Rahbarizadeh et al. 2011). While CDR domain swapping can decrease the binding ability of nanobodies (Hwang and Foote 2005; Rahbarizadeh et al. 2011), utilization of scaffolds with specific properties is an attractive option for improving or preparing functional proteins. The amino acid sequence of the scaffold is highly conserved across nanobodies (Conrath et al. 2001; Saerens et al. 2005; Deschaght et al. 2017), which means that only small number of residues need to be modified.

In the present study, we used *P. pastoris* cells for the production of chimeric nanobodies to test the possibility of CDR swapping. We utilized green fluorescent protein (GFP)-nanobody (Rothbauer et al. 2006) and cluster of differentiation 4 (CD4)-nanobody (Roobrouck and Stortelers 2015) as models for preparing chimeric nanobodies. GFP-nanobody is one of the most popular nanobodies with the crystal structure of which has been analyzed (Kubala et al. 2010). CD4 is a cell surface antigen of mammalian immune cells that can be used as a marker for predicting clinical outcome in colorectal tumor (Galon et al. 2006). Unlike GFP-nanobody, the crystal structure of CD4-nanobody has not yet been resolved. Here we demonstrate methods for easy production of chimeric nanobodies in *P. pastoris* cells by utilizing seamless cloning. We also tested the production of chimeric nanobodies tagged with long peptide sequences for easy purification and detection of nanobodies.

## Materials and methods

### Strains and media

Cells and media were prepared according to the previous report (Aoki et al. 2011). *E. coli* strain DH5α [F^−^, Φ80d *lacZ*ΔM15, Δ(*lacZYA–argF*)U169, *deoR*, *recA*1, *endA*1, *hsdR*17(r_K_^–^ m_K_^+^), *phoA,* *supE*44, λ^–^, *thi*–1, *gyrA*96, *relA*1] (Toyobo, Osaka, Japan) was used as a host for plasmid construction. *P. pastoris* (*Komagataella phaffii*) strain GS115 [*his4*] was purchased from Thermo Fisher Scientific (Rockford, IL). *E. coli* cells transformed with plasmids were grown on lysogeny broth (LB) medium [0.5% (w/v) yeast extract, 1% (w/v) Bacto Tryptone, 1% (w/v) NaCl] containing 100 μg/mL ampicillin. After transformation with plasmids, *P. pastoris* cells were grown on minimal dextrose (MD) agar plate containing 1.34% (w/v) yeast nitrogen base w/o a.a., 2% (w/v) glucose, and 2% (w/v) agar. Obtained *P. pastoris* colonies were precultured in buffered glycerol complex (BMGY) medium containing 1% (w/v) yeast extract, 2% (w/v) peptone, 100 mM KPO_4_ pH 6.0, 2.68% (w/v) yeast nitrogen base w/o a.a., and 0.2% 500× biotin. After preculture, cells were grown in a buffered methanol complex (BMMY) medium (expression medium) containing 1% (w/v) yeast extract, 2% (w/v) peptone, 100 mM KPO_4_ pH 6.0, 2.68% (w/v) yeast nitrogen base w/o a.a., 0. 2% 500× biotin, and 0.5% (v/v) methanol.

### Plasmid construction and transformation

**Fig. 1:**
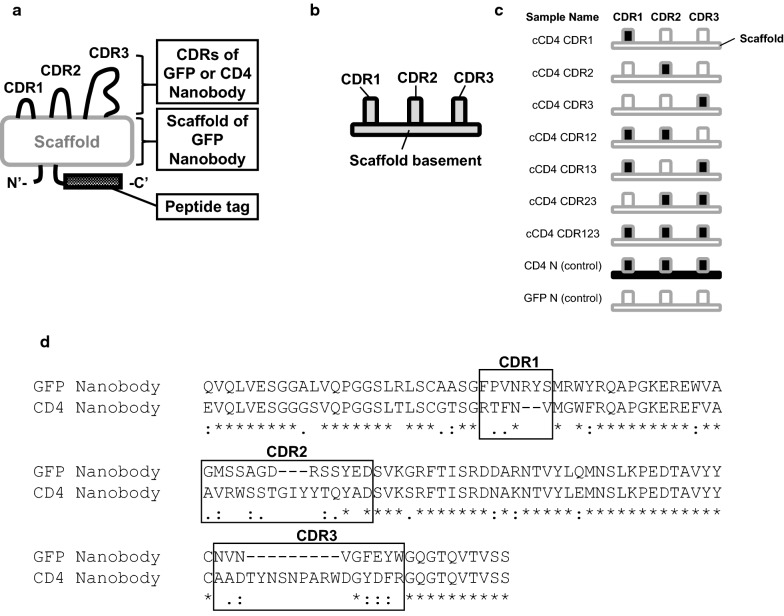
Chimeric nanobodies prepared in the present study. **a** Construction of chimeric nanobodies. Peptide tags were fused to the C-terminus of nanobodies. **b** Simple illustration of a nanobody used for swapping. **c** Name and construction of chimeric nanobodies. Long white block: nanobody scaffolds with amino acid sequences derived from GFP-nanobody, long black block: nanobody scaffolds with amino acid sequences derived from CD4-nanobody, small white block: CDRs with amino acid sequences derived from GFP-nanobody, small black block: CDRs with amino acid sequences derived from CD4-nanobody, cCD4 CDRX: chimeric nanobody with CDRX of GFP-nanobody replaced with that of CD4-nanobody, CD4N: CD4 -nanobody with peptide tags containing two FLAG-tag sequences (see also Additional file 1: Table S3), GFPN: GFP-nanobody with peptide tags containing two FLAG-tag sequences (see also Additional file 1: Table S3). **d** Amino acid sequence alignment of GFP- and CD4-nanobodies prepared by CLUSTALW (Larkin et al. 2007). Asterisks indicate identical amino acids. Colons indicate similar amino acids. Dots indicate almost similar amino acids. Blanks indicate non-homologous amino acids

**Fig. 2:**
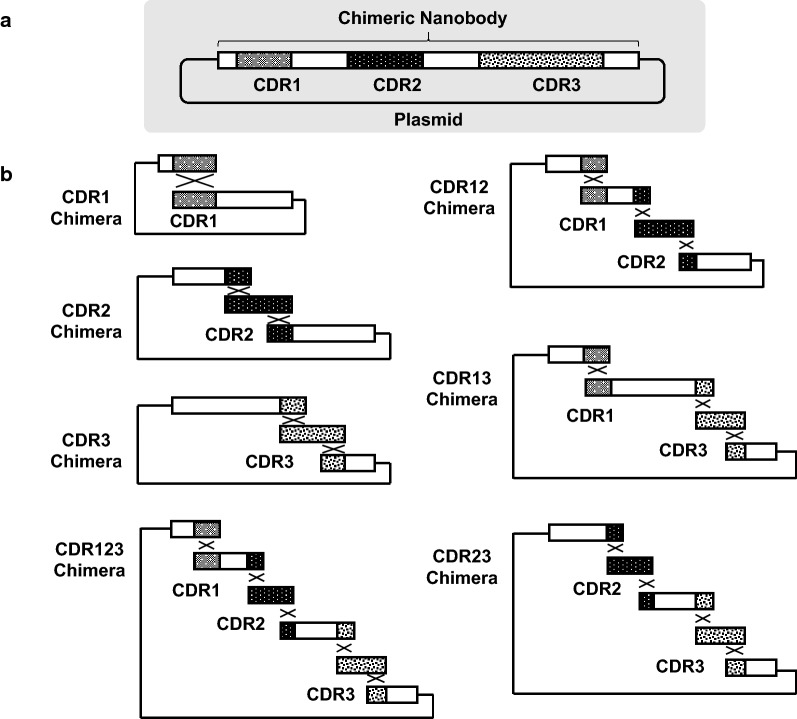
Overview of plasmid construction schemes for production of chimeric nanobodies. **a** Overview of the composition of chimeric nanobodies. Nanobodies with CDR1, 2, and 3 were inserted into single plasmids. **b** Scheme for preparing each chimera. Cross: positions of homologous recombination

All primers used in this study are listed in Additional file 1: Table S1. Nucleotide sequences encoding GFP-nanobody (Rothbauer et al. 2006) and CD4-nanobody (Roobrouck and Stortelers 2015) genes were optimized for expression in *P. pastoris*, as shown in Additional file 1: Table S2, and were purchased from Integrated DNA Technologies, Inc (Coralville, IA). Chimeric nanobodies were constructed as shown in Fig. 1. Both nucleotide sequences for GFP-nanobody and CD4-nanobody were submitted to the DDBJ Sequenced Read Archive with accession numbers LC472971 and LC472972, respectively. Nucleotide fragments were amplified by KOD plus Neo DNA polymerase (Toyobo, Osaka, Japan). DNA fragments were integrated into a pPIK9K vector (Invitrogen) using an In-Fusion HD Cloning Kit (Takara Bio, Otsu, Japan) to produce recombinant proteins with the amino acid sequences shown in Additional file 1: Table S3. For each nanobody, long peptide tags containing two FLAG-tag coding sequences were added to the C-terminus for easy handling of nanobodies (Fig. 1a). Figure 2 shows the types of nucleotide fragments prepared for the construction of each chimeric nanobody. Nucleotide sequence of the plasmids were determined by Sanger sequencing (Eurofins Genomics, Tokyo, Japan). The plasmids were digested with *Sac*I (Toyobo) and transformed to *P. pastoris* cells using Frozen EZ Yeast Transformation II Kit (Zymo Research, Irvine, CA), following manufacturer’s instructions.

### Protein production and purification

Secreted recombinant proteins were purified with minor modifications to previously reported methods (Aoki et al. 2011; Miyamoto et al. 2019). To produce nanobodies, *P. pastoris* colonies on MD agar plate were cultured in 20 mL of BMGY medium with shake at 250 rpm, 48 h at 30 °C. The cells were cultured in 15 mL of BMMY medium for 24 h at 30 °C with shake at 250 rpm. Production of recombinant proteins were checked by using 10 μL of culture supernatants for SDS-PAGE as described in the next section. Cell culture was transferred to a 50 mL tube and centrifuged at 8000×*g* for 60 min at 4 °C. Culture supernatants (15 mL) obtained after centrifugation were filtrated with 0.22 μm filter and concentrated in a Amicon Ultra-15 Centrifugal Filters Ultracel-10K (Millipore, Billerica, MA). The samples were washed with TBS buffer (Nacalai, Kyoto, Japan) twice and resuspended in TBS buffer. The obtained sample solution (5 mL) containing FLAG-tagged recombinant proteins were purified by 250 μL of anti-FLAG M2 affinity gel (Sigma, Saint Louis, MO) using disposable columns, following manufacturer’s instructions. Proteins were eluted from the affinity gel using 1 mL of 0.1 M glycine–HCl (pH 3.5) and immediately neutralized by mixing with 20 μL of 1 M Tris (pH 8.0). The protein solution was added to Amicon Ultracel-3K (Millipore) and the buffer was replaced three times with 500 μL of PBS. The obtained protein solution was kept at 4 °C until use. The final protein concentration in the samples was determined by SDS-PAGE as described below.

### SDS-PAGE

Culture supernatants, purified proteins, or a serial dilution of bovine serum albumin (BSA) (225, 450, 900, 1125, and 2250 ng per lane) were loaded into e-PAGEL 5–20% (Atto, Tokyo, Japan) and stained with CBB Stain One Super (Nacalai) according to the manufacturer’s instructions. Gel images were obtained using the ImageQuant LAS 4000 minisystem (GE Healthcare Bio-Sciences AB, Uppsala, Sweden) and analyzed with ImageJ software (Abramoff et al. 2004). The protein concentration was quantified by applying the band intensity to a standard curve generated from the analysis of protein bands prepared using BSA.

### Obtaining binding abilities of nanobodies by surface plasmon resonance

Recombinant GFP (ProSpec, Ness-Ziona, Israel) or CD4 (R&D Systems, MN, USA) were immobilized on a Sensor Chip CM5 (GE Healthcare) using an Amine Coupling Kit (GE Healthcare) according to the manufacturer’s instructions for the BIACORE-T200 instrument (GE Healthcare). CD4 (20 μg/mL, pH 6.0) and GFP (20 μg/mL, pH 5.0) solutions were prepared using 10 mM HEPES buffer (GE Healthcare) or 10 mM acetate buffer (GE Healthcare), respectively. CD4 or GFP were immobilized on CM5 by an automatic program according to the manufacturer’s instructions at 25 °C with a flow rate of 10 μL/min. The binding of nanobodies to CD4 or GFP was tested at 25 °C according to the manufacturer’s instructions for the BIACORE-T200 instrument using HBS-EP buffer (0.01 M HEPES pH 7.4, 0.15 M NaCl, 3 mM EDTA, and 0.005% (w/v) Surfactant P20) as a running buffer. Nanobodies were prepared to a concentration of 0.4 μM using HBS-EP buffer. Binding to CD4 and GFP was analyzed under the following conditions: contact time 180 s, flow rate 30 μL/min, and dissociation time 180 s. Sensor chips were recovered by 10 mM NaOH with a contact time of 30 s and a flow rate of 30 μL/min. Response units of CD4- or GFP-nanobodies (CD4 N and GFP N, respectively) to CD4 or GFP, respectively, were used as positive controls. All the data obtained were analyzed by Biacore T200 Evaluation Software (GE Healthcare) and the binding levels (increase of relative response from the base line) were obtained. The data were adjusted as follows. For testing binding abilities of nanobodies to CD4, the relative binding level of CD4 N was set to 1. For testing binding abilities of nanobodies to GFP, the relative binding level of GFP N was set to 1. Statistical significance was evaluated using the unpaired Student’s *t* test for comparisons between two means. For all experiments, significance was established at p ≤ 0.05.

## Results

### Production of recombinant nanobodies by *P. pastoris*

**Fig. 3:**
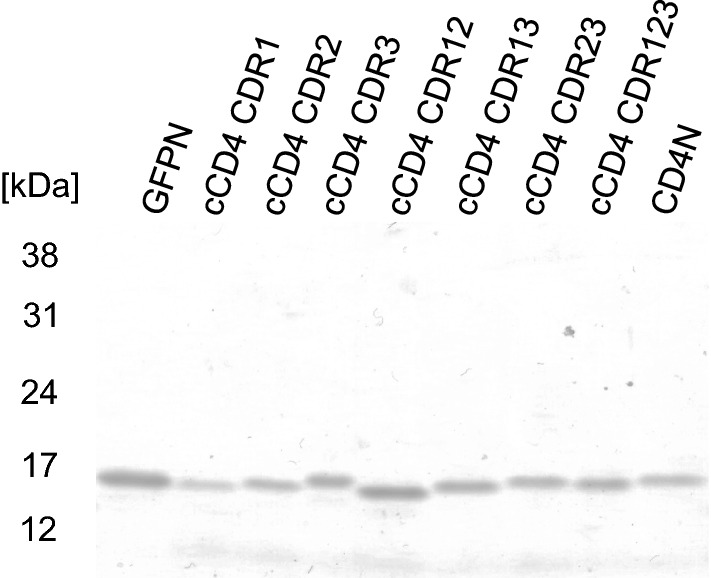
Chimeric nanobodies detected in the culture supernatants of *P. pastoris* transformants. Culture supernatants of transformants (10 μL for each) were loaded. GFP N and CD4 N represents GFP- or CD4-nanobodies, respectively, tagged with long peptide tags containing two FLAG-tags

Chimeric nanobodies (Fig. 1) were successfully constructed by one-step cloning of 1–4 nucleotide fragments (Fig. 2). Recombinant proteins in 10 μL *P. pastoris* culture supernatant showed clear single bands (Fig. 3). The molecular masses of each band ranged from 14 to 17 kDa, representing the molecular weight of the products.

### Relative binding abilities of chimeric nanobodies to CD4

**Fig. 4:**
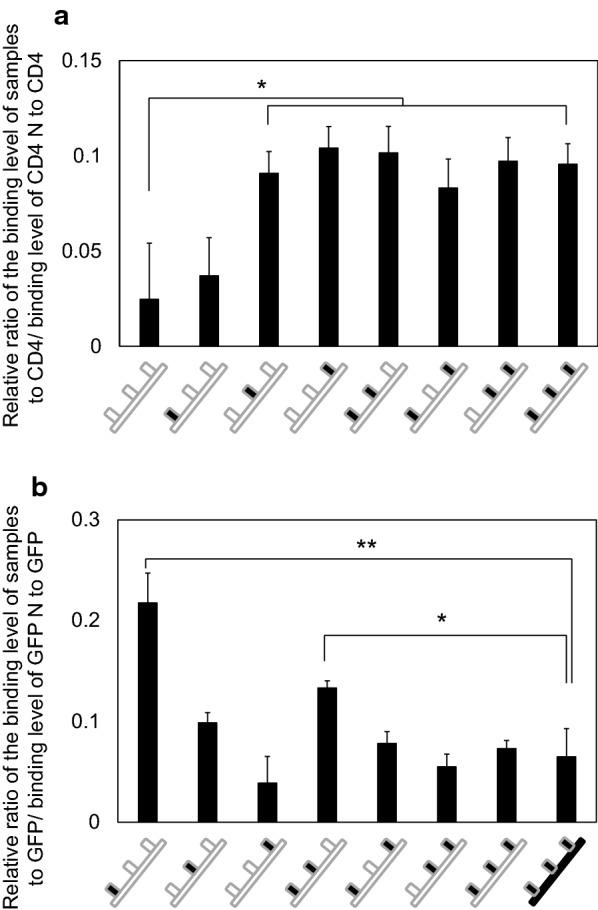
Relative binding abilities of chimeric nanobodies to CD4 (**a**) and GFP (**b**). See also Fig. 1c for details of the illustration. Long block: nanobody scaffolds, small block: CDRs, white block: amino acid sequences derived from GFP-nanobody, black block: amino acid sequences derived from CD4-nanobody. **a** Relative response of chimeric nanobodies to CD4 compared to the response of CD4-nanobody. See also Additional file 1: Table S4 for the binding level in each experiment before normalization. **b** Relative response of chimeric nanobodies to GFP compared to the response of GFP-nanobody. See also Additional file 1: Table S5 for the binding level in each experiment before normalization. N = 3, bar = mean ± SD, **p < 0.01, *p < 0.05

CD4 and GFP were immobilized onto a CM5 chip at 9695.5 response unit (RU) and 1189.8 RU, respectively. Both nanobodies showed specific binding to their original targets (CD4 and GFP, respectively). CD4- and GFP-nanobodies did not respond to non-target molecules (GFP and CD4, respectively) (data not shown). To test the effect of CDR swapping on the ability of the nanobodies to bind to novel targets, relative binding abilities of chimeric nanobodies to CD4 was investigated (Fig. 4a and Additional file 1: Table S4). The response of GFP-nanobody (GFP N) to CD4 [0.025 ± 0.029 when response of CD4-nanobody (CD4N) to CD4 was set to 1] was approximately 40 times lower than that of CD4-nanobody. There were no significant changes when the CDR1 of GFP-nanobody was replaced with that of CD4-nanobody (0.037 ± 0.020). On the other hand, when the CDR2 (0.091 ± 0.011) or CDR3 (0.10 ± 0.011) of GFP-nanobody was replaced with that of CD4, the chimeric nanobodies showed a significant increase in relative binding response to CD4 compared to the original GFP-nanobody. There were no significant differences in response to CD4 among the chimeric nanobodies in which CDR2 or 3 is replaced, suggesting the importance of CDR2 and 3 in CD4 binding. Chimeric nanobodies in which all CDRs had been replaced showed approximately 10% response to CD4-nanobody (0.096 ± 0.011).

### Relative binding abilities of chimeric nanobodies to GFP

To test the effect of CDR swapping of original binding targets, the binding abilities of chimeric nanobodies to GFP was investigated (Fig. 4b). The response of CD4-nanobody to GFP [0.065 ± 0.028 when response of GFP-nanobody (GFP N) to GFP was set to 1]was approximately 15 times lower than that of GFP-nanobody. When the CDR1 of GFP-nanobody was replaced with that of CD4, the relative binding response to GFP dropped by 80% (0.22 ± 0.029), although this was still a significantly higher response than with the CD4-nanobody. When the CDR3 of GFP-nanobody was replaced with that of CD4-nanobody, the relative binding response to GFP (0.039 ± 0.026) was similar to that of CD4-nanobody (0.065 ± 0.028), suggesting that the replacement of CDR3 results in loss of the ability to bind to GFP.

## Discussion

The present study demonstrated a simple and easy way of producing chimeric nanobodies in *P. pastoris* using one-step cloning of up to four nucleotide fragments. Since primer sequences used for preparing CDR and scaffold fragments are easily randomized or mutated, this method could be useful in producing large-scale libraries of chimeric nanobodies for high-throughput screening, including screening methods utilizing peptide-barcode (Egloff et al. 2019; Miyamoto et al. 2019). Notably, this method can be also used together with other synthetic methods for nanobody preparation (Moutel et al. 2016), which would enable easy preparation of nanobodies with variety of CDR length. In the chimeric nanobodies produced in the present study, replacement of CDRs significantly altered the response of nanobodies to both GFP and CD4, as expected. Replacement of the scaffold caused a decrease in the binding ability of nanobodies to targets, which is comparable to previous reports (Hwang and Foote 2005; Rahbarizadeh et al. 2011; Zabetakis et al. 2013). Scaffolds of GFP- and CD4-nanobodies have seven similar, three almost similar, and three non-homologous amino acid residues (Fig. 1d), suggesting that the decreased response to substrates is dependent on these amino acid residues. Unexpectedly, we did not detect significant synergistic increase in relative binding abilities among CDRs of CD4 nanobody on the scaffold of GFP nanobody. It is possible that synergistic effect of CDRs do not always happen; it has been previously reported by Zabetakis and colleagues that that synergistic increase in thermostability did not occur when CDRs of nanobody against the toxin Staphylococcal enterotoxin B was swapped by different CDRs (Zabetakis et al. 2013). Further investigation is needed to determine how the specific CDRs and scaffolds synergistically or non-synergistically affect binding abilities of nanobody with respect to polypeptide length and diverse sequences. Nevertheless, this study demonstrated design and high-throughput production of various chimeric nanobodies using *P. pastoris*. Future studies will focus on randomization of CDRs in addition to three non-homologous amino acid residues on scaffolds to obtain novel and highly specific nanobodies via high-throughput screening.

## Additional file

**Additional file 1.** Additional tables.

## Data Availability

All relevant data are within the manuscript and its additional files. Nucleotide sequence data reported are available in the DNA Data Bank of Japan (DDBJ, http://www.ddbj.nig.ac.jp) under the Accession Numbers LC472971 and LC472972.
